# Metabolic Syndrome and Its Associated Factors among Type 2 Diabetic Patients in Southwest Ethiopia, 2021/2022

**DOI:** 10.1155/2022/8162342

**Published:** 2022-09-20

**Authors:** Dereje Gemeda, Endegena Abebe, Abdissa Duguma

**Affiliations:** ^1^Department of Biomedical Sciences, College of Health Sciences, Mettu University, Mettu, Ethiopia; ^2^Department of Nursing, College of Health Sciences, Mettu University, Mettu, Ethiopia

## Abstract

**Background:**

Metabolic syndrome is one of the leading global public health and clinical challenges, with increasing prevalence figures ranging from 10% to as high as 84%. There is a 5-fold increase in metabolic syndrome associated with type 2 diabetes and a 2-fold increase associated with the development of cardiovascular disease. The calorie-dense diet and sedentary life patterns are the most important contributory factors that have been established.

**Objective:**

This study is aimed at assessing metabolic syndrome and its associated factors among patients with type 2 diabetes attending chronic follow-up units at hospitals in Southwest Ethiopia, in 2020.

**Methods:**

This study was conducted at hospitals in Southwest Ethiopia, using an institutional-based cross-sectional study design from September 1, 2020, to August 30, 2021. Data was collected from 422 type 2 diabetics through face-to-face interviews using structured and pretested questionnaires. Data were entered into Epi-data version 4.4.1 and exported for analysis to the Statistical Package for Social Sciences 25. The magnitude of metabolic syndrome was determined by using the National Cholesterol Education Program Third Adult Treatment Panel III criteria. Bivariate and multivariable logistic regression analyses were used to evaluate associated factors for the outcome variable. A *P* value of less than 0.05 was considered statistically significant. *Result and Conclusion*. Out of the 422 sample sizes, 394 type 2 diabetics participated in this study, giving a response rate of 93.36%. The overall prevalence of metabolic syndrome among type 2 diabetics in this study was 68.3%, using the National Cholesterol Education Program and Third Adult Treatment Panel criteria. The associated factors for metabolic syndrome among type 2 diabetics are urban residency, old age, physical inactivity, salt intake, inadequate fruit and vegetable consumption, palm oil, and eating meat.

## 1. Background

Metabolic syndrome (MetS) is an aggregate of interconnected physiological and biochemical factors that directly increase the risk of atherosclerotic cardiovascular disease. The main components of metabolic syndrome include abdominal obesity, high blood pressure, high blood triglycerides, low levels of high-density lipoprotein cholesterol, and hyperglycemia [1–5].

According to the Global Burden of Disease Study, high total cholesterol levels caused 44.4 million deaths and 938 million DALYs in 2016, making them the seventh and eighth highest risk factors for women and men, respectively, in terms of attributable DALYs [4].

Hypertension is estimated to cause 7.5 million deaths worldwide, which is about 12.8% of the total of all deaths. This accounts for 57 million disability-adjusted life years (DALYs) or 3.7% of total DALYs [2]. Diabetes had a global 22.9 million incidence, 476.0 million prevalence, 1.37 million death, and 67.9 million disability-adjusted life-years (DALYs) in 2017, with projections of 26.6 million incidences, 570.9 million prevalence,1.59 million death, and 79.3 million disability-adjusted life-years in 2025 [3].

About 35% of men and women in the United States have MetS. A systematic analysis conducted in various European countries found that the prevalence of MetS in type 2 diabetes ranged from 3% to 71.7%. It has previously been suggested that MetS is present in about 13–30% of people in developing countries [6–9]. According to a systematic analysis, the prevalence of MetS in the Asia-Pacific region in 2007 was in the range of 11.9 to 37.1 percent [8]. In sub-Saharan Africa (SSA), a systematic review found that the average approximate prevalence of MetS ranged from 24 to 90.6% among type 2 diabetics [9, 10]. Based on the National Cholesterol Education Program Third Adult Treatment Panel (NCEP: ATP III) criteria, the prevalence of MetS among type 2 diabetics in Ethiopia was between 26% and 70.1% [11, 12].

Each year, at least 2. 8 million people die as a result of being overweight or obese, with overweight or obesity accounting for 35.8 million (2.3 percent) of global DALYs. Evidence has shown that almost 70-80% of the DM population has been diagnosed with MetS [13]. It has been shown that the presence of MetS in type 2 diabetic patients reduces the survival rate by at least 10 years [14]. MetS has a 5 times higher risk of developing type 2 diabetes and 2-3 times higher risk of developing CVD over the next 5 to 10 years [15].

A systematic review and meta-analysis revealed that the highest prevalence of MetS among type 2 diabetics in SSA countries was observed in Ethiopia [16]. The prevalence of MetS among type 2 diabetics in Ethiopia grew from 45.6% to 70.3% in 2016 [11, 12].

The epidemiological transition, in which the burden of chronic disorders like metabolic syndrome is rising, is currently occurring in developing countries. To date, no research has been done to demonstrate the prevalence of metabolic syndrome in a study setting. There are some new variables which were incorporated in this study: stress, duration of sleep, and eating meat. Therefore, this study was intended to assess the magnitude of MetS and its associated factors among patients with type 2 diabetes in Southwest Ethiopia.

## 2. Materials and Methods

### 2.1. Study Area and Period

This study was conducted at Mettu Karl Referral Hospital, Bedele General Hospital, Didessa Primary Hospital, and Darimu Primary Hospital, which are located in the Southwest part of Ethiopia.

The study was carried out from 01 September 2020 to 01 August 2021.

### 2.2. Study Design

This is an institutional-based cross-sectional study design.

### 2.3. Source Population

The source population is all type 2 diabetic patients attending chronic follow-up units at hospitals in Ilu Ababor and Buno Bedele.

### 2.4. Study Population

All sampled type 2 diabetic patients attending chronic follow-up units at hospitals in Ilu Ababor and Buno Bedele were available during the data collection period.

### 2.5. Inclusion Criteria

The inclusion criteria are those type 2 diabetics with age greater than or equal to 18 years.

### 2.6. Exclusion Criteria

The exclusion criteria are the critically ill patients and pregnant woman.

### 2.7. Sample Size Determination

The sample size was calculated by using a single population proportion formula.

The number of samples required for this study was calculated by using single proportion formula with an assumption of 95% confidence interval and 5% of marginal error, where the standard normal deviation is 1.96 and the prevalence (*p*) of MetS among type 2 diabetics was 51.1%, taken from Ayder Comprehensive Specialized Hospital, Tigray [7].

(1) $$\begin{array}{c} n=\frac{\left({\left(Z\alpha /2\right)}^{2}P\left(1-p\right)\right)}{d^{2}},\\ \;n=\frac{\left(1.96\right)\;2*0.511\left(0.489\right)\;}{\left(0.05\right)\;2}=383.96=384. \end{array}$$

We took maximum sample size, and by adding 10% nonresponse rate, our final sample size is 422. Finally, 10% of the nonresponses rate was added yielding the final sample size of 422.

### 2.8. Sampling Technique and Procedure

**Figure 1:**
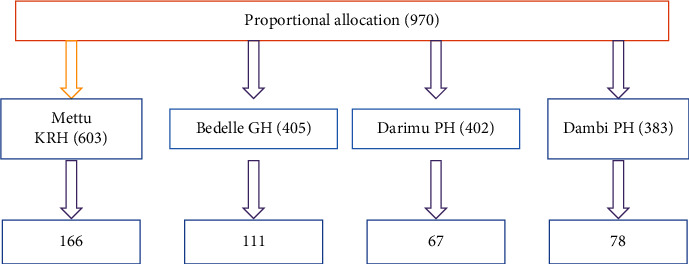
Proportional allocation of sample size for assessment of MetS and its associated factors among type 2 diabetics, 2020/2021.

Simple sampling technique was used to select type 2 diabetics. The total number of type 2 diabetic patients attending chronic follow-up units for similar 45 days of the last year at Mettu Karl Referral Hospital, Bedele General Hospital, Darimu, and Dambi Primary Hospitals was 603, 405, 402, and 280, respectively. Based on their proportion every four intervals, the participants were selected from all hospitals (Figure 1). The first study participant was selected randomly and continued until the desired sample size was achieved.

### 2.9. Data Collection and Processing

Trained data collectors record the data. A virtual sphygmomanometer (with an ok size) and a stethoscope have been used to measure blood pressure. A BP was taken, while the study subject is in a sitting position, from the arm with the higher value after the patient rested for 5 minutes before beginning measurement. Three measurements of BP (mmHg) on a single visit were taken, spaced 1-2 minutes apart, and the averages of at least two records were used for the computation of results.

A simple flexible metric tape calibrated in centimeters was used for measuring waist circumference. It was measured midway between the iliac crest and the lower rib margin in the horizontal plane while the participant was standing with his/her arms at the sides, feet positioned close together. The measurement was taken at the end of normal expiration to the nearest 0.1 centimeters.

Five milliliters of venous blood was collected from each patient. The extracted serum was investigated for HDL-C and triglyceride (in mg/dl) using an automated clinical chemistry analyzer following the manufacturer's instructions. Fasting blood glucose (FBS) measurement was taken after overnight fasting (≥8 hours), and plasma glucose (in mg/dl) was determined using a glucose blood monitor (AccuChek, China).

### 2.10. Study Variables

#### 2.10.1. Dependent Variable

The dependent variable is metabolic syndrome.

#### 2.10.2. Independent Variables

The independent variables are as follows: age, sex, place of residence, occupational status, educational level, duration of diabetes, type of treatment, history of chronic disease(s), family history of diabetes, smoking cigarettes, drinking alcohol, physical activity, stress, duration of sleep, frequency of fruit and vegetable intake, type of oil used, amount of salt intake, and frequency of eating meat.

### 2.11. Operational Definitions and Definition of Terms

*Obesity*: abnormal or excessive fat accumulation that presents a risk to health. A body mass index (BMI) over 25 is considered overweight, and over 30 is Obase[2].

*Dyslipidemia*: a high level of lipids in the blood (e.g., triglycerides, high-density lipoprotein cholesterol, and low-density lipoprotein cholesterol). HDL cholesterol is made up of a lot of protein. HDL-C is a “good cholesterol” because it prevents plaque formation, collects extra cholesterol and fat, has antioxidant properties, protects against thrombosis, maintains endothelial function, and keeps blood viscosity low. Fat cells constitute triglycerides, which can be broken down and used to provide energy for the body's metabolic processes, including growth [2–4].

*Hyperglycemia*: a circumstance wherein there is abnormally excessive glucose in the blood plasma.

*Level of stress*: Perceived Stress Scale contains 10 questions with each alternative answer: 0, never; 1, also rarely–sometimes; 3, fairly often; and 4, very often. First, reverse your scores for questions 4, 5, 7, and 8. On these four questions, change the scores like this: 0 = 4, 1 = 3, 2 = 2, 3 = 1, and 4 = 0. Individual scores range from 0 to 40 with higher scores indicating higher perceived stress.

*Stress*: scores ranging from 0 to 13.

*Moderate stress*: scores ranging from 14 to 26.

*High-perceived stress*: scores ranging from 27 to 40.

*Physically active*: patient who did vigorous plus moderate physical activity.

*Vigorous physical activity*: includes plowing, cutting crops, gardening (digging), grinding, laboring, loading furniture, instructing spinning (fitness), cycle driving, soccer, tennis, and fast swimming. A person meeting any of the following criteria is classified in this category:

- Vigorous intensity activity on at least 3 days, achieving a minimum of at least 1,500 MET-minutes/week OR

*Moderate physical activity*: cleaning, washing, gardening, hand milking cows, planting and harvesting crops, digging dry soil, weaving, woodwork, mixing cement, laboring, walking with a load on the head, and drawing water are all examples of manual labor. A person not meeting the criteria for the “high” category but meeting any of the following criteria is classified in this category: -3 or more days of vigorous intensity activity of at least 20 minutes per day OR -5 or more days of moderate intensity activity or walking of at least 30 minutes per day.

*Low physical activity*: a person not meeting the criteria of moderate and vigorous physical activity.

### 2.12. Data Processing and Analysis

The data was coded and entered into Epi-data version 4.4.1 before being exported to the Statistical Program for Social Sciences (SPSS) version 25 software package for analysis. The magnitude of MetS was determined according to the NCEP: ATP III criterion. Modified NCEP/ATP III criterion was used to diagnose metabolic syndrome. Study participants were classified as having MetS if they have three or more of the following parameters met: waist circumference (male ≥ 102 cm and female ≥ 88 cm), low HDL cholesterol (<40 mg/dl for male and <50 mg/dl for female), elevated triglyceride concentration (≥150 mg/dl), raised BP (≥130/85 mmHg or on treatment), and elevated FBS (≥110 mg/dl or on treatment) [6].

The bivariate logistic regression model was used to see the association between the dependent variable and the independent variable. Variables with a *p* value ≤ 0.25 on bivariate analysis were entered into a multivariable logistic regression model to identify factors that independently predict the MetS after controlling potential confounding effects. All standard error of each variable and variance inflation factor (VIF) were checked, and finally, multivariable logistic regression was conducted to control confounding factor. The strength of association between the different associated factors and the study outcomes was reported using adjusted odds ratios, and the presence of a statistically significant association was considered at alpha less than or equal to 0.05. Continuous variables were expressed as their mean with standard deviation, while categorical variables were expressed as a proportion.

### 2.13. Data Quality Control

The questionnaire was prepared in English and was translated to Amharic and Afan Oromo by language experts then back to English in these languages to ensure consistency of the meaning. Before starting the data collection, a pretest was conducted on 5% of the total sample size at Jimma Medical Center and modification was considered accordingly. We gave training to the data collectors and supervisor regarding the purpose of the study, measurement techniques, and ethical issues for 2 days. The authors provided regular supervision to monitor the work. The proper functioning of instruments, laboratory reagents, and technical performance was checked based on the manufacturer's manual by using quality control samples. The authors examined all collected data for completeness and consistency during data management, storage, and analysis.

### 2.14. Ethical Consideration

We carried out this study after obtaining ethical approval from the Institutional Review Committee of the College of Health Science, Mettu University, and obtained informed consent from each participant before being included in the study, including the benefits and risks. A blood sample was collected (5 ml) after explaining the importance of the study and the risk of having blood drawn from the left arm, including mild pain when the needle goes in. We kept patients' confidentiality throughout the study, and the patient has the right to withdraw at any time during the study.

## 3. Results

### 3.1. Sociodemographic Characteristics of the Respondents

Out of the 422 sample sizes, 394 type 2 diabetic patients participated in this study, giving a response rate of 93.36%. About 56.1% of respondents were males by sex. Regarding age, about 31% were found between 41 and 50, and more than two-thirds (68%) of study participants were living in urban areas. Concerning the marital status, about 76.5 of them got married, and regarding ethnic status, the majority of them were Oromo, which accounted for 68%. In terms of educational level, the majority of them, which is about 37.8% of the study participants, were unable to read and write, and the majority of them were merchants, which accounts for 27.6% (Table 1).

### 3.2. Prevalence of Metabolic Syndrome

**Figure 2:**
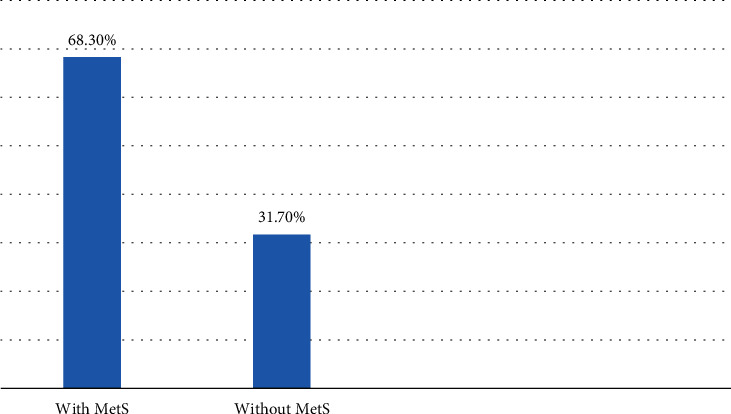
Metabolic syndrome of type 2 diabetic patients at hospitals in Ilu Aba Bora and Buno Bedele zones, Southwest Ethiopia, 2020/2021.

The overall prevalence of MetS among type 2 diabetic patient in this study was 68.3% using NCEP/ATP III criteria (Figure 2).

### 3.3. Factors Affecting Metabolic Syndrome among Type 2 Diabetic Patients

#### 3.3.1. Multivariable Logistic Regression

In multivariable logistic regression, residence, eating fruit, eating vegetables, physical activity, and salt intake were variables which were significantly associated with metabolic syndrome. Regarding age, being in the 51–64 age group was AOR 9.00 [2.19, 36.93] times more likely to have metabolic syndrome compared to the age group between 18 and 25. In the case of fruit intake, those individuals consuming fruit less than twice a week were AOR 4.68 [1.58, 13.79] times more likely to have metabolic syndrome as compared to those consuming more than twice a week. Concerning vegetable intake, those who consumed vegetables less than twice a week were 5.09 [1.85, 14.0] times more likely to have metabolic syndromes compared to those who did not consume vegetables more than twice a week. In case of physical activity, being physically active was AOR 6.93 [2.58, 18.62] times more likely to have MetS as compared to being physically active. Those individuals using palm oil were 5.56 [2.03, 15.24] times more likely to have MetS as compared to nonusers. Individuals who ate more than two meals per week were more likely to have MetS than those who ate less than two meals per week (Table 2).

## 4. Discussion

This study is aimed at assessing the prevalence of metabolic syndrome and its associated factors among type 2 diabetic patients attending chronic follow-up units at hospitals in Mettu and Bedele zones, Southwest Ethiopia.

The metabolic syndrome is a collection of physiological and biochemical variables that work together to raise the risk of atherosclerotic cardiovascular disease. The metabolic syndrome is characterized by abdominal obesity, high blood pressure, high blood triglycerides, low levels of HDL cholesterol, and hyperglycemia [1].

From 422 sampled population, 394 participated, giving 93.37% response rate. The overall prevalence of MetS among type 2 diabetic patients in this study was 68.3% using NCEP/ATP III criteria, which lies within a range of 12–86% from a review study done in sub-Saharan Africa [5]. But, this study is higher than a study conducted in Hawassa (45.9%) [6], in Tigray (51.1%) [7], in 10 European countries (24%), and in the UK (32%) [8]. This difference is due to differences in the study setting, sample size, and the criteria standard IDF used to define metabolic syndrome, respectively.

The findings of this study are comparable with the findings of the study conducted in Ghana (68.6%) [9] and Nigeria (68.7%) [10]. The magnitude of MetS in this study is lower than that in studies conducted in Gondar (70.3%) [11] and Iran (64.9%) [12]. This variation could be due to differences in the sociocultural background, study setting, and lifestyle.

This study reveals that older age (51-64) was significantly associated with metabolic syndrome, and this is in line with a study conducted in Tigray [7] and in Iran [12]. This association might be due to older age participants' being physically inactive and adopting unhealthy lifestyles.

This study reveals that those respondents who ate fruits and vegetables once a week and never were significantly associated with metabolic syndrome when compared to those who ate fruit and vegetables twice and more a week, and this is supported by EPHA (Ethiopia) [17] and is in line with the study conducted in Tigray [7] and Brazil [18]. One possible explanation is that fruits and vegetables comprise relatively lower energy and higher fiber [13], antioxidant content [14], and lower glycemic index [15].

Participants who were not physically active were 6.9 times more likely to have MetS as compared to those who were physically active. This is in line with studies done in Tigray [7], sub-Saharan Africa [19], and the USA [20]. The effects of physical activity could be explained by its beneficial effects on body composition, including improving skeletal muscle insulin sensitivity and reducing insulin resistance [16].

Respondents who used palm oil in food were 5.5 times more likely to have MetS when compared to those who used natural sunflower oil. This may be due to palm oils having more saturated fat, which may increase dyslipidemia. Respondents who ate meat twice or more a week were 5.9 times more likely to have MetS when compared to those who ate meat once a week and never.

### 4.1. Conclusion and Recommendations

#### 4.1.1. Conclusion

The magnitude of metabolic syndrome among type 2 diabetes mellitus is 68.3%. The associated factors for metabolic syndrome among type 2 diabetics are urban residence, old age, physical inactivity, salt intake, inadequate fruit and vegetable consumption, palm oils, and eating meat.

#### 4.1.2. Recommendations

Based on the above findings, the following recommendations have been forwarded.

- The regional health office has to provide health education on diet intake based on the national protocol
- The zonal and woredas sports offices have to organize the place, time, and situation favorable for doing physical exercise

## Figures and Tables

**Table 1:** Sociodemographic characteristics of type 2 diabetic patients at hospitals in Southwest Ethiopia, 2020/2021.

Variables		No.	Percent
Sex	Female	173	43.9
	Male	221	56.1

Age	≤30	75	19
	31-40	83	21
	41-50	125	31.7
	51-64	111	28.3

Residence	Urban	268	68
	Rural	126	32

Marital status	Single	32	8.1
	Married	302	76.5
	Divorced	33	8.4
	Widowed	27	7

Ethnic	Oromo	248	63
	Amhara	102	25.8
	Other^1^	44	11.2

Educational level	Cannot read and write	149	37.8
	<Primary school	88	22.4
	<High school	22	5.5
	College/university	103	26.1
	Postgraduate degree	32	8.2

Occupational status	Farmer	71	18
	Merchant	109	27.6
	Gov't employee	80	20.3
	Private employee	34	8.6
	Housewife	71	17.9
	Other^2^	29	7.6

^1^Gurage and Tigre; ^2^Daily laborer.

**Table 2:** Multivariable logistic regression analysis of factors associated with metabolic syndrome among patients with type 2 diabetics at hospitals in Southwest Ethiopia.

Variables	Category	MetS		COR	AOR
		Yes	No		
Residence	Rural	102	24	1	1
	Urban	167	101	2.57 [2.102, 5.32]	0.298 [0.090, 0.987]^∗^

Age	18-30	31	44	1	1
	31-40	43	40	0.757 [0.479, 6.791]	1.804 [0.479, 6.791]
	41-50	96	29	0.212 [0.905, 7.649]	3.246 [0.905, 11.649]
	51-64	99	12	0.085 [1.195, 19.931]	9.003 [2.195, 36.931]^∗^

Smoking status	Never smoke	150	107	1	1
	Ever smoke	84	53	0.884 [0.212, 1.671]	0.947 [0.312, 2.872]

Alcohol intake	Never drink	75	69	1	1
	Ever drink	156	94	0.654 [0.124, 0.026]	0.677 [0.224, 2.046]

Fruit intake	>Twice a week	100	83	1	1
	<Twice a week	201	10	0.059	4.681 [1.589, 13.794]^∗^

Vegetable intake	>Twice a week	99	84	1	1
	<Twice a week	194	17	0.068 [2.851, 10.032]	5.095 [1.852, 14.018]^∗^

Physical activity	Active	98	62	1	1
	Inactive	212	22	0.306 [2.434, 11.522]	6.938 [2.585, 18.623]^∗^

Salt intake	<1 teaspoon per/day	157	5	1	1
	>1 teaspoon per a day	213	9	1.326 [1.93, 11.344]	7.194 [2.393, 21.624]^∗^

Type treatment	*Insulin*	98	51	1	1
	By OHGAs	144	101	1.353 [0.239, 1.242]	0.398 [0.139, 1.143]

Duration of sleep	>8 hrs	99	94	1	1
	≤8 hrs	104	97	0.982 [0.123, 1.354]	0.436 [0.169, 1.12]

Palm oil used	Not used	174	7	1	1
	Used	196	17	2.155 [1.13, 12.348]	5.567 [2.03, 15.248]^∗^

Frequency of eating meat	<Twice a week	165	10	1	1
	>Twice a week	201	18	1.477 [2.246, 12.223]	5.993 [2.246, 15.990^∗^]

Notes: ^∗^significant at *p* value < 0.05.

## Data Availability

We can affirm that the data we have used for this research is available on our hands, and we can provide it when it is needed by this journal.

## Abbreviations

AOR: Adjusted odds ratio
BMI: Body mass index
BP: Blood pressure
COR: Crude odds ratio
EDHS: Ethiopian Demography Health Survey
OHGAs: Oral hypoglycemic agents
OR: Odds ratio
SDG: Sustainable Development Goal
SPSS: Statistical Package for Social Sciences
W/H2: Weight over height square
WHO: World Health Organization.
